# Curdlan oligosaccharides induce salicylic acid-dependent defense responses in *Arabidopsis thaliana*: structural characteristics and immune priming

**DOI:** 10.3389/fbioe.2026.1831123

**Published:** 2026-05-25

**Authors:** Chuntian Liu, Chen Zhao, Le Su, Qiulin Yue, Kunlun Li, Lei Sun, Qun Su, Marisol Freire Seijo, Yelenys Alvarado Capó, Xin Sun, Li Tian, Song Zhang, Lin Zhao

**Affiliations:** 1 State Key Laboratory of Biobased Material and Green Papermaking, School of Bioengineering, Qilu University of Technology, Shandong Academy of Sciences, Jinan, China; 2 Jinan Hangchen Biotechnology Co., Ltd., Jinan, China; 3 Shandong Baoyuan Biotechnology Co., Ltd., Yantai, China; 4 Plant Biotechnology Institute, Central University “Marta Abreu” of Las Villas, La Provincia de Villa Clara, Santa Clara, Cuba

**Keywords:** curdlan oligosaccharides, defense enzymes, plant immunity, ROS burst, salicylic acid pathway

## Abstract

**Introduction:**

Long-term use of chemical pesticides has caused environmental and resistance-related concerns, highlighting the need for environmentally friendly plant immune inducers. This study aimed to prepare curdlan oligosaccharides (CDOS) and evaluate their immune-inducing activity in Arabidopsis thaliana.

**Methods:**

CDOS were prepared by enzymatic hydrolysis and purified by dialysis. Their structural characteristics were analyzed by TLC, FTIR, and ESI-MS. Antibacterial activity against Pseudomonas syringae pv. tomato DC3000 (Pst DC3000), disease resistance in A. thaliana, ROS accumulation, defense-related enzyme activities, and immune-related gene expression were further investigated.

**Results:**

The purified CDOS preparation mainly consisted of oligosaccharides with a degree of polymerization of 2–3, with a total sugar content of 81.62% and a residual protein content of 3.25%. CDOS showed no direct antibacterial activity against Pst DC3000 but significantly enhanced resistance in A. thaliana, reducing bacterial colonization by approximately 50% at 50 mg/L. CDOS also promoted ROS accumulation, increased POD, SOD, CAT, and PAL activities, and upregulated the SA-associated marker genes PR1, PR2, and PR5, whereas the JA-associated genes VSP and PDF1.2 were not significantly altered. The protective effect was impaired in SA-pathway-deficient mutants but retained in the JA-pathway mutant.

**Discussion:**

These findings indicate that CDOS functions primarily as a plant immune elicitor rather than a direct antibacterial agent, and its protective activity is closely associated with SA-dependent defense responses.

## 1 Introduction

Modern agriculture has long relied on chemical pesticides, resulting in biodiversity loss, soil and water contamination, and increased pathogen resistance, all of which have gradually reduced the effectiveness of conventional pesticides (Hua et al., 2024; Hou et al., 2025). Against this backdrop, plant immune inducers (PIIs) have emerged as a novel approach in agricultural disease management. These inducers activate the plant innate immune system without directly killing pathogens, thereby reducing environmental damage and the risk of pathogen resistance development (Zhang et al., 2023; Qiu et al., 2017). In response to the increasing severity of agroecological challenges, the development of such environmentally friendly immune inducers has become increasingly important for sustainable agriculture.

A class of oligosaccharide elicitors, such as chitooligosaccharides, laminarin, and CDOS (Zhang et al., 2023), function by mimicking PAMPs/DAMPs and being recognized by membrane-bound PRRs, thereby activating pattern-triggered immunity (PTI). This process triggers rapid signaling events—including calcium influx, MAPK phosphorylation, and a reactive oxygen species burst—and primes plants into a “sensitized” state (Chaube et al., 2022; Rey et al., 2018), enabling a stronger defense response upon pathogen infection. Key characteristics of this state include upregulation of pathogenesis-related (PR) genes, enhanced phytoalexin biosynthesis, and callose deposition. Such immune pre-activation confers broad-spectrum systemic acquired resistance (SAR) while avoiding the growth penalties associated with constitutive defense activation, thereby offering an environmentally compatible strategy for disease control (Fu and Dong, 2013). Although these biocompatible elicitors capable of regulating multiple defense signaling nodes have attracted growing interest, their complex chemical structures and high preparation costs continue to significantly hinder research progress.

CDOS, which are functional degradation products of microbially derived β-1,3-glucan (curdlan), have attracted considerable attention due to their ability to efficiently activate plant immunity (Tang et al., 2019). This abundant microbial origin also ensures a sustainable supply for the preparation of these oligosaccharides (Melo et al., 2020; Fu et al., 2015). Li et al. found that CDOS effectively activates the innate immune system in potatoes. It triggers robust early and late defense responses at both biochemical and proteomic levels, successfully reducing the disease area of late blight by approximately 50%. Furthermore, the induced resistance was short-lived and had no adverse effects on potato yield, demonstrating its potential as an environmentally friendly plant protection agent (Li et al., 2014). However, a major challenge remains: the signaling pathways of CDOS and their practical efficacy under complex field conditions are still not well understood, which severely hinders their practical application.

To address the pressing challenges of chemical pesticide residues and pathogen resistance in plant protection, this study aims to develop a novel environmentally friendly plant immunity elicitor by preparing CDOS through enzymatic hydrolysis and systematically investigating their function and mechanism in activating systemic plant immunity. This research employs enzymatic hydrolysis to prepare CDOS and further investigates their immune-inducing activity in the *Arabidopsis thaliana*-Pst DC3000 pathosystem. The novelty of the present study lies not merely in preparing CDOS, but in evaluating non-derivatized enzymatically generated curdlan oligosaccharides in a genetically tractable model system and clarifying that their protective effect is associated with host immune activation and SA-related signaling rather than direct antibacterial activity. These findings provide new insights for the development of novel biopesticides and environmentally friendly disease-control strategies.

## 2 Materials and methods

### 2.1 Preparation of CDOS

#### 2.1.1 Enzymatic hydrolysis

The crude enzyme solution was prepared by fermentation of the laboratory-preserved strain A-B17-1. CDOS was produced via enzymatic hydrolysis by adding curdlan powder to the crude enzyme solution as a well-dispersed suspension at a substrate concentration of 1% (w/v), followed by incubation at 28 °C for 2 h under continuous shaking. After enzymatic hydrolysis, the reaction mixture was centrifuged to collect the supernatant. The supernatant was subsequently concentrated and stored at 4 °C for subsequent use.

#### 2.1.2 Purification of CDOS

The impurity proteins were removed by the Sevag method. Briefly, the Sevag reagent was prepared at a volume ratio of n-butanol:chloroform = 1:4 (v/v). The reagent was then mixed with the oligosaccharide solution at a 1:4 (v/v) volume ratio, followed by vigorous shaking for 20 min and subsequent centrifugation at 8,000 rpm for 10 min. The upper aqueous phase was aspirated and collected, and the aforementioned operation was repeated until no protein layer was observed between the aqueous and organic phases. After completion of deproteinization, vacuum concentration was conducted to eliminate residual reagents (Qu et al., 2013; Shi et al., 2019).

Inorganic salts were removed by dialysis. Briefly, a dialysis bag with a molecular weight cutoff (MWCO) of 200 Da was first boiled in deionized water for 2 min and then removed to cool to room temperature. One end of the dialysis bag was clamped, the enzymatic hydrolysate was loaded through the other end, and the opening was then sealed with another clip. After confirming no leakage from the dialysis bag, it is immersed in deionized water, and the deionized water is replaced every 2 h. Upon completion of dialysis, the oligosaccharide solution is freeze-dried and stored at 4 °C for subsequent use.

#### 2.1.3 Determination of degradation rate and oligosaccharide yield

To evaluate the efficiency of CDOS preparation, the degradation rate of curdlan and the sugar-based oligosaccharide yield were determined. After enzymatic hydrolysis, the reaction mixture was centrifuged, and the insoluble residue was collected, washed with deionized water, dried to constant weight, and recorded as *W*_*r*_. The degradation rate was calculated as follows:

$$\text{Degradation rate }\left(\%\right)=\left[\left(W_{0}\;‐\;W_{r}\right)\;/\;W_{0}\right]\times 100$$

where *W*_*0*_ is the initial dry weight of curdlan and *W*_*r*_ is the dry weight of the insoluble residue after enzymatic hydrolysis.

After deproteinization, dialysis, and freeze-drying, the dry weight of the recovered product was recorded as *W*_*p*_. Based on the measured total sugar content of the freeze-dried product (*C*_*s*_), the oligosaccharide yield was calculated as follows:

$$\text{Oligosaccharide yield }\left(\%\right)=\left[\left(W_{p}\times C_{s}\right)\;/\;W_{0}\right]\times 100$$

where *W*_*p*_ is the dry weight of the freeze-dried product, *C*_*s*_ is the total sugar content expressed as a decimal fraction, and *W*_*0*_ is the initial dry weight of curdlan.

### 2.2 Structural analysis

#### 2.2.1 Thin layer chromatography

Thin-layer chromatography (TLC) was employed for oligosaccharide analysis, utilizing silica gel GF 254 plates as the stationary phase and a mixture of 1-butanol/acetic acid/water (2:2:1, v/v/v) as the mobile phase (Reiffová and Nemcová, 2006). After separation, oligosaccharide spots were visualized by spraying the TLC plate with aniline-diphenylamine-phosphoric acid reagent and subsequent heating at 110 °C for 5–10 min. Monosaccharide glucose and disaccharide lactose were used as reference standards.

#### 2.2.2 Fourier-transform infrared

The infrared spectra of CDOS and their corresponding polysaccharides were detected using a PerkinElmer Spectrum Two infrared spectrometer. A small amount of sugar solid powder was placed on the optical path of the sample stage, with a blank used as the background. The scanning wavelength range was 4,000–500 cm^-1^, and the scanning resolution was set to 4 cm^-1^ (Wang et al., 2022).

#### 2.2.3 Electrospray ionization mass spectrometry

The obtained CDOS were analyzed by electrospray ionization time-of-flight mass spectrometry (ESI-TOF-MS) under the following conditions: the ion source was an electrospray ionization source, operating in positive ion mode. The nitrogen temperature was set at 325 °C with a flow rate of 10 L/min, and the nebulizer pressure was 30 psig. The capillary voltage was 4 kV, collision voltage was 135 V, and Octopole RF Peak was 750 V. The mass acquisition range was m/z 50–3,000 Da (Allison et al., 2020).

### 2.3 Antibacterial activity of CDOS against Pst DC3000

The bacteriostatic activity of CDOS against *Pseudomonas syringae* pv. tomato DC3000 (Pst DC3000) was assessed by measuring inhibition zone diameters. CDOS solutions were prepared as concentration gradients of 25, 50, 100, and 200 mg/L by re-dissolving freeze-dried CDOS powder in sterile deionized water, and were then sterilized by filtration through 0.22 μm microporous membranes. Sterile deionized water was therefore used as the corresponding vehicle control. Then, 100 μL of each concentration was added to Oxford cups on agar plates previously inoculated with Pst DC3000. Simultaneously, blank control (100 μL sterile water) and positive control (100 μL kanamycin sulfate solution) were established. All plates were incubated in a constant temperature incubator at 28 °C for 24 h, with each experimental group performed in triplicate (Zhang et al., 2019). The inhibition-zone diameter was recorded as the full diameter of the clear zone across the Oxford cup.

### 2.4 Plant growth and pathogen inoculation

The wild-type *A*. *thaliana* was purchased from Beijing Huayueyang Biotechnology Co., Ltd. Mutant lines sid2, NahG, and jar1 were kindly provided by Researcher Heng Yin from the Dalian Institute of Chemical Physics, Chinese Academy of Sciences. *A*. *thaliana* seeds were soaked in a small amount of deionized water and vernalized at 4 °C for 72 h. Subsequently, the seeds were inoculated onto MS agar medium plates and cultivated in a controlled environment (temperature: 22 °C, humidity: 70%, photoperiod: 12 h light/12 h dark). When the seedlings reached the two-true-leaf-and-one-cotyledon stage, they were transplanted into a soil mixture (black soil:vermiculite = 1:2.5, v/v) and grown for an additional 4 weeks. Finally, 4-week-old *A. thaliana* plants were selected for experimental use. *Pseudomonas syringae* pv*. tomato* DC3000 (Pst DC3000) was cultured in King’s B medium (containing 10 g/L protease peptone, 1.5 g/L K_2_HPO_4_, and 15 mL/L glycerol) with shaking at 180 rpm at 28 °C. The concentration of the bacterial suspension was adjusted to an OD_600_ of approximately 0.5–1.0 using 10 mM magnesium sulfate solution.

### 2.5 Bacterial quantification

Bacterial quantification was employed to investigate the activation effect of oligosaccharide pretreatment on the immune response of *A. thaliana (A. thaliana)* and its inhibitory effect on pathogen infection. Brief Procedure: Leaf samples were collected (3 leaves per plant) and surface-sterilized with 75% ethanol, followed by three rinses with sterile water. The leaves were homogenized in 10 mmol/L MgSO_4_ solution. The homogenate was serially diluted to 10^–4^ and 10^–5^, and 100 µL of each dilution was plated on King’s B agar plates containing 50 μg/mL rifampicin. After incubation at 28 °C for 48 h, bacterial colonies were counted. The experiment was independently repeated three times (Zhang et al., 2019).

### 2.6 Treatment and disease assessment

CDOS solution was prepared at 50 mg/L concentration by re-dissolving freeze-dried CDOS powder in sterile deionized water, with 50 mg/L chitosan oligosaccharide solution as positive control and sterile deionized water as blank control. This working concentration was selected based on the preliminary concentration-screening assay (25, 50, 100, and 200 mg/L; see Figure 3b), in which 50 mg/L showed the strongest reduction in bacterial colonization. These solutions were sprayed onto 4-week-old wild-type and mutant *A. thaliana* plants exhibiting optimal growth. Each treatment was performed with three biological replicates, each containing five plants. Three days after spraying, 50 μL of bacterial suspension or magnesium sulfate solution was infiltrated into rosette leaves using a needleless syringe. Disease severity was quantified using a four-tiered scale based on necrotic lesion area: Level 1 (0%–25%), Level 2 (25%–50%), Level 3 (50%–75%), and Level 4 (75%–100%) (Wang et al., 2022). The disease index was calculated as:

$$\text{Disease index }\left(\%\right)=\left(\sum \left(\text{ Disease level}\times \text{Number of leaves per level}\right)/\text{Total leaves}\times \text{the highest level }\right)\times 100$$

### 2.7 Reactive oxygen species (ROS) detection

Rosette leaves were collected from 4-week-old *A. thaliana* plants with good growth status. The lower epidermis of the leaves was carefully peeled off using forceps, then placed in MES/KCl buffer (10 mM MES/KOH, 50 mM KCl, 100 µM CaCl_2_, pH 6.5) and stabilized for 30 min under light conditions. Subsequently, the lower epidermis was transferred to a 50 µM 2′,7′-dichlorodihydrofluorescein diacetate (H_2_DCFDA) dimethyl sulfoxide (DMSO) solution and incubated for 30 min in the dark. Excess probe was then washed away with Tris/KCl buffer, followed by incubation in an oligosaccharide solution for 10 min. Observation and photography were performed using a fluorescence microscope at an excitation wavelength (Ex) of 420–485 nm and an emission wavelength (Em) of 515 nm (Zhao et al., 2023).

### 2.8 Enzyme activity assays

Approximately 0.1 g of *A. thaliana* leaf tissue was homogenized in 1 mL of extraction buffer on ice for 2 min. The homogenate was then centrifuged at 8,000 *g* and 4 °C for 10 min. The resulting supernatant was collected and kept on ice for subsequent analysis. The activities of peroxidase (POD) and superoxide dismutase (SOD) and catalase (CAT), as well as the phenylalanine ammonia-lyase (PAL), were quantified using commercial assay kits (Solarbio, China) in accordance with the standardized protocols. For defense-enzyme assays, 50 mg/L CDOS was used as the working concentration because this concentration showed the strongest protective effect in the preliminary pathogen-resistance assay.

### 2.9 qPCR analysis of immune-related genes

*Arabidopsis thaliana* wild-type and mutant plants were processed for transcript level and defense enzyme activity analyses using the following protocol. Curdlan oligosaccharide solution was prepared at 50 mg/L concentration by re-dissolving freeze-dried CDOS powder in sterile deionized water, with sterile deionized water serving as the blank control. This working concentration was selected based on the preliminary concentration-screening assay, in which 50 mg/L showed the strongest reduction in bacterial colonization. The solutions were applied by spraying to 4-week-old Arabidopsis plants exhibiting optimal growth. At 24 h post-application, complete plant specimens were collected and flash-frozen in liquid nitrogen, then maintained at −80 °C for subsequent analysis.

Total RNA was extracted from *A. thaliana* leaves using the Accurate SteadyPure Plant RNA Extraction Kit (Takara Plant RNA Kit). RNA purity (OD_260_/OD_280_ ratio) was verified with a NanoPhotometer N60 ultramicro spectrophotometer from Implen. Reverse transcription was performed using the ABconal reverse transcription kit (ABScript III RT Master Mix for qPCR with gDNA Remover). Quantitative real-time PCR analysis was conducted using a Rotor-Gene Q real-time PCR instrument from Qiagen (Germany) and the ABconal qPCR kit (2X Universal SYBR Green Fast Qpcr Mix) under the following conditions: pre-denaturation at 95 °C for 5 min; followed by 40 cycles of denaturation at 95 °C for 30 s, annealing at 58 °C for 30 s, and extension at 72 °C for 30 s. Primer specificity was verified by melt curve analysis, and relative gene expression levels were calculated using the 2^^−ΔΔCt^ method.

### 2.10 Statistical analysis

One-way ANOVA was performed, followed by Duncan’s multiple-comparison test, utilizing SPSS software (version 26). A p-value <0.05 was considered statistically significant.

## 3 Results

### 3.1 Physicochemical and structural analysis

**FIGURE 1:**
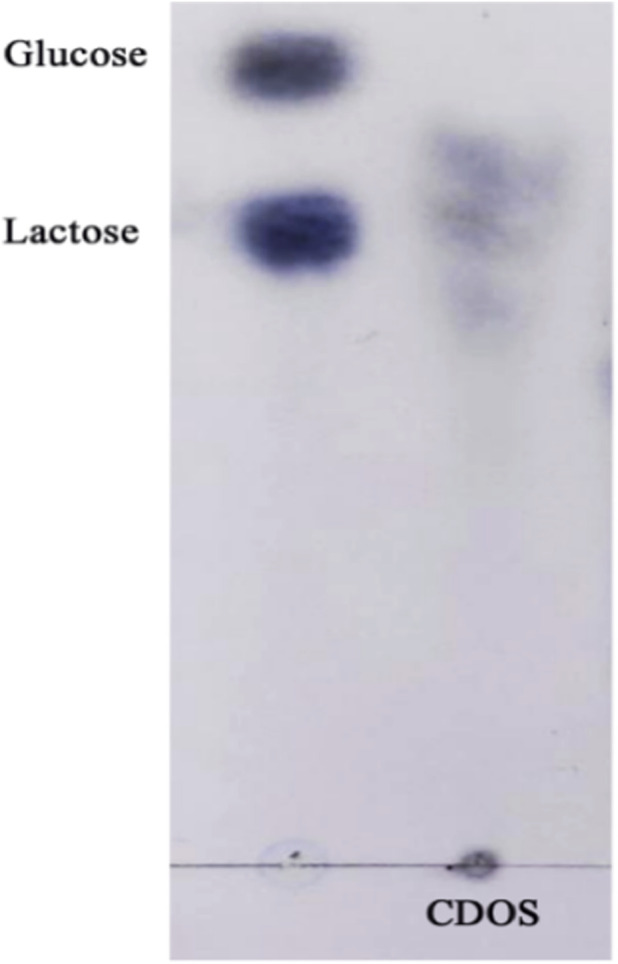
TLC analysis of CDOS.

Analysis revealed that, after enzymatic hydrolysis and subsequent purification, the degradation rate of curdlan was 31%, and the oligosaccharide yield was 23%. The purified CDOS preparation, following deproteinization and desalting, contained 3.25% residual protein and 81.62% total sugar. As shown in Figure 1, TLC analysis suggests that the curdlan oligosaccharide mixture likely consists of di- and/or trisaccharides.

**FIGURE 2:**
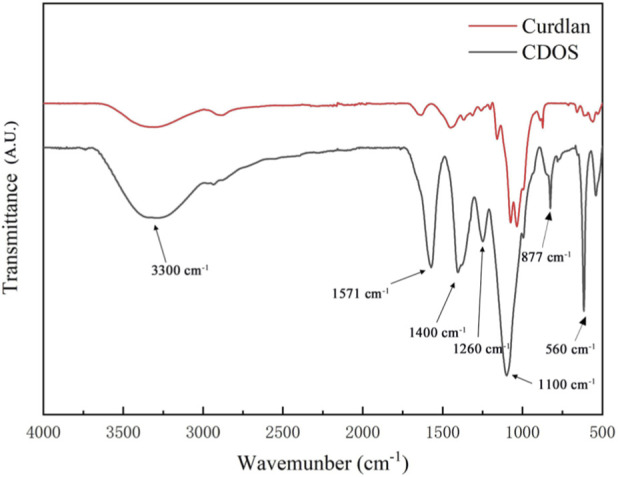
FTIR spectra of curdlan and CDOS.

The structures of curdlan and its oligosaccharides were characterized using Fourier Transform Infrared (FTIR) spectroscopy. As shown in Figure 2, compared to native curdlan, the characteristic absorption peaks of its degradation product, CDOS, at 3,000 cm^-1^ (O-H stretching vibration), 1,260 cm^-1^ (C-H bending vibration), and 1,100 cm^-1^ (secondary alcohol group vibration) all exhibited a systematic red shift. This phenomenon indicates that the cleavage of the sugar chains disrupts the intramolecular hydrogen bonding network, significantly altering the chemical environment and electron density of the functional groups, thereby reducing their vibrational energy levels.

Further analysis of the absorption changes at specific wavenumbers revealed that the characteristic peak near 877 cm^-1^, attributed to β-glycosidic bonds, was significantly weakened in CDOS, providing direct evidence for the cleavage of glycosidic linkages within the polysaccharide chain. Concurrently, the absorption peak near 560 cm^-1^, associated with pyranose ring skeletal vibrations, also showed noticeable displacement and shape changes, supporting the structural remodeling from the perspective of conformational alterations. Collectively, this spectroscopic evidence confirms that the degradation process successfully produced CDOS through the cleavage of glycosidic bonds, resulting in fundamental changes to its molecular structure.

The electrospray ionization mass spectrometry (ESI-MS) results are summarized in Table 1. Analysis of the mass-to-charge ratios (m/z) indicated that the CDOS preparation mainly consisted of oligosaccharides with DP 2–3, with DP2 and DP3 as the predominant detected components. Specifically, the major ions detected corresponded to the sodium adduct of DP2 at m/z 365.11, the potassium adduct of DP2 at m/z 381.80, and the sodium adduct of DP3 at m/z 527.16. These results indicate that enzymatic hydrolysis of curdlan generated a CDOS preparation enriched in low-degree-of-polymerization oligosaccharides.

**TABLE 1:** ESI-MS characterization of the degree of polymerization of CDOS.

DP	Observed value m/z	Calculated value m/z	Predicted value m/z	Molecular formula	Error	Peak intensity (a.u.)
2	365.1061	365.1061	365.106	[C_12_H_22_O_11_+Na]^+^	0.00027	8,213.7
2	381.7971	381.7971	381.3978	[C_12_H_22_O_11_ + K]^+^	1.05	6664.91
3	527.1625	527.1625	527.1582	[C_18_H_32_O_16_+Na]^+^	0.0082	1706.15

### 3.2 CDOS-mediated disease resistance in *A. thalian*a

**FIGURE 3:**
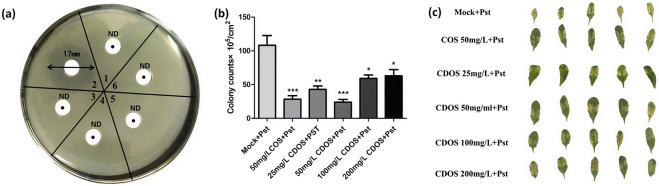
CDOS-mediated resistance of *Arabidopsis thaliana* to Pst DC3000. **(a)** *In vitro* antibacterial assay of CDOS against Pst DC3000. 1, sterile deionized water; 2, kanamycin sulfate; 3-6, CDOS at 25, 50, 100, and 200 mg/L, respectively. The inhibition-zone diameter was measured as the full clear-zone diameter across the Oxford cup. ND indicates no measurable inhibition zone. **(b)** Bacterial colonization of Pst DC3000 per cm^2^ of leaf tissue (*P < 0.05, **P < 0.01, ***P < 0.005). **(c)** Representative lesion phenotypes of *Arabidopsis thaliana* leaves after inoculation with Pst DC3000.

The disc diffusion assay showed that only the positive control, kanamycin sulfate, produced a measurable inhibition zone (17 mm), whereas sterile water and all tested CDOS concentrations showed no measurable inhibition zone (Figure 3a). These results indicate that CDOS did not exhibit direct antibacterial activity against Pst DC3000 under the present assay conditions, suggesting that its protective effect is associated with activation of host immune responses rather than direct inhibition of the pathogen. Three days prior to inoculation with Pst DC3000, *A. thaliana* was pretreated with CDOS at concentrations ranging from 25 to 200 mg/L. The result revealed that CDOS pretreatment significantly reduced the bacterial load in leaves, with the 50 mg/L concentration exhibiting the most pronounced effect, resulting in an approximate 50% reduction compared to the control group (Figure 3b). Representative lesion phenotypes are shown in Figure 3c. Consistent with the bacterial colonization results, CDOS-pretreated leaves exhibited attenuated disease symptoms, and the 50 mg/L treatment showed the most evident protective phenotype. Therefore, 50 mg/L was used as the working concentration in the subsequent mechanistic analyses.

### 3.3 CDOS-induced plant immunity in mutant *Arabidopsis thaliana*

**FIGURE 4:**
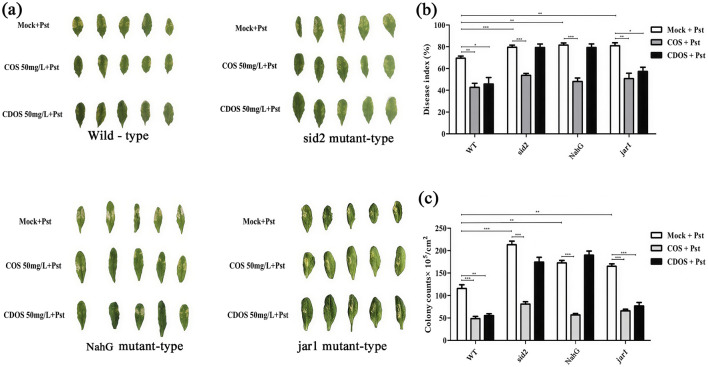
CDOS-mediated resistance of different *Arabidopsis thaliana* genotypes to Pst DC3000. **(a)** Phenotypic symptoms of infected leaves. **(b)** Disease index quantification. **(c)** Bacterial colonization of Pst DC3000 per cm^2^ of leaf tissue (*P < 0.05, **P < 0.01, ***P < 0.005).

A study on disease resistance signaling pathways in different *Arabidopsis* mutants (Figure 4) showed that under untreated conditions, the disease indices of salicylic acid pathway-deficient mutants (sid2, NahG) and the jasmonic acid pathway-deficient mutant (jar1) increased by 10.00%, 12.00%, and 11.33%, respectively, compared to the wild-type, confirming the important roles of these two pathways in basal resistance. After oligosaccharide treatment, the disease indices of wild-type plants were significantly reduced by 27.00% and 24.00% following COS and CDOS treatments, respectively. In the jar1 mutant, COS and CDOS also reduced the disease indices by 30.33% and 23.67%, respectively. However, in the sid2 and NahG mutants, COS treatment still reduced the disease indices by 26.00% and 33.67%, respectively, while CDOS treatment did not cause a significant reduction.

These results indicate a fundamental difference in the signaling pathways activated by the two oligosaccharides to elicit immune responses: the disease resistance effect of CDOS strictly depends on an intact SA signaling pathway, as its efficacy is completely lost when this pathway is impaired; whereas the effect of COS does not rely on the JA pathway and maintains significant activity even when the SA pathway is partially compromised, suggesting it may trigger immune responses by preferentially activating the SA pathway or other pathways independent of JA.

### 3.4 ROS induction by CDOS

**FIGURE 5:**
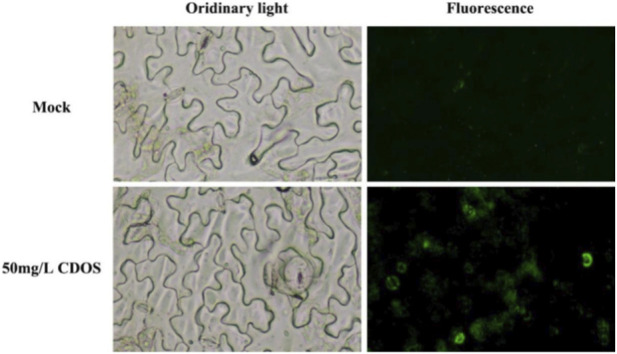
CDOS-induced ROS production in *Arabidopsis thaliana* epidermal cells. ROS accumulation was detected using the fluorescent probe H_2_DCFDA (green).

As shown in Figure 5, only faint fluorescence was observed in the untreated control group, indicating a low basal level of ROS; in contrast, intense green fluorescence was detected in CDOS-treated leaves, suggesting that CDOS promotes ROS accumulation in *A. thaliana* tissues. These fluorescence-based results support the involvement of ROS-related early signaling in CDOS-induced immune responses.

### 3.5 CDOS modulates defense enzyme dynamics in *Arabidopsis thaliana* immunity

**FIGURE 6:**
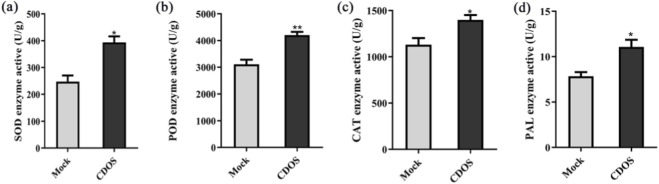
Defense-related enzyme activities in CDOS-treated plants. **(a)** superoxide dismutase (SOD) activity. **(b)** peroxidase (POD) activity. **(c)** catalase (CAT) activity. **(d)** phenylalanine ammonia-lyase (PAL) activity. Data represent mean ± SD (*P < 0.05, **P < 0.01).

In this study, *A. thaliana* was treated with CDOS at the selected working concentration of 50 mg/L for 24 h, and the subsequent activity changes of four defense enzymes—POD, SOD, CAT, and PAL—were investigated. As shown in Figure 6, following CDOS treatment, POD activity increased by 35.20%, SOD activity by 59.01%, CAT activity by 23.80%, and PAL activity by 41.18%. These results indicate that CDOS may enhance lignin synthesis in *A. thaliana* by modulating the activities of PAL and POD, thereby contributing to improve disease resistance in the plant. Additionally, the increased activities of SOD and CAT are likely to mitigate oxidative stress responses in *A. thaliana*, protecting plant cells from oxidative damage.

### 3.6 Effects of CDOS pretreatment on the expression of resistance-related genes in mutant *Arabidopsis thaliana*

**FIGURE 7:**
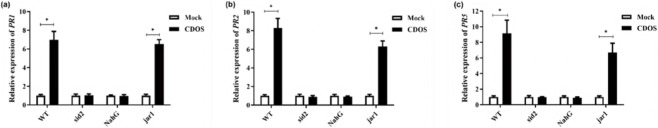
Activation of SA pathway marker genes by CDOS. Relative expression levels (fold change) of the SA-responsive genes **(a)** *PR1*, **(b)** *PR2*, and **(c)** *PR5* were quantified by qRT-PCR after CDOS pretreatment in the indicated genotypes (*P < 0.05).

**FIGURE 8:**
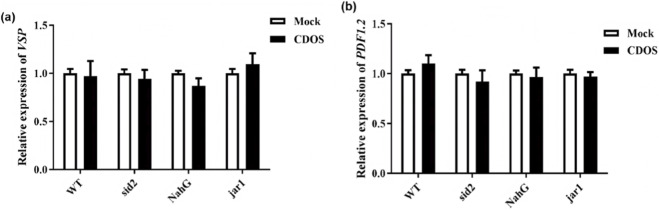
Relative expression levels (fold change) of the JA-responsive genes **(a)** VSP and **(b)** PDF1.2 were quantified by qRT-PCR after CDOS pretreatment in the indicated genotypes.

The results of gene expression changes are shown in Figures 7, 8. In wild-type plants, the relative expression levels of the salicylic acid pathway marker genes *PR1*, *PR2*, and *PR5* increased to 6.97-, 8.30-, and 9.15-fold relative to the mock-treated control, respectively, whereas the jasmonic acid pathway marker genes *PDF1.2* and *VSP* showed no significant changes. In salicylic acid pathway mutants, no notable expression changes were observed in *PR1*, *PR2*, or *PR5*, and similarly, *VSP* and *PDF1.2* also remained unchanged. In the jasmonic acid pathway mutant, the relative expression levels of *PR1*, *PR2*, and *PR5* increased to 6.85-, 7.12-, and 7.76-fold relative to the mock-treated control, respectively, whereas *VSP* and *PDF1.2* still exhibited no significant alterations. CDOS pretreatment preferentially induces SA-associated defense responses, and this response does not depend on an intact JA pathway but requires functional SA signaling. However, because central regulatory nodes such as *NPR1* were not examined in the present study, the SA signaling chain cannot yet be considered fully resolved.

## 4 Discussion

In this study, CDOS were successfully prepared by enzymatic hydrolysis of curdlan, with a degradation rate of 31% and a sugar-based oligosaccharide yield of 23%. Structural analyses indicated that the purified product was enriched in low-degree-of-polymerization oligosaccharides, mainly with DP 2–3, and contained 81.62% total sugar with 3.25% residual protein. The combined TLC, FTIR, and ESI-MS data support that the obtained product was generated through cleavage of β-glycosidic linkages in curdlan and that the resulting structural changes were associated with the formation of bioactive low-DP oligosaccharides (Chen et al., 2015; Kong, 2007).

Although CDOS did not exhibit direct antibacterial activity against Pst DC3000 *in vitro*, they significantly enhanced resistance in *Arabidopsis thaliana*, reducing bacterial colonization in leaves by approximately 50% at the selected working concentration of 50 mg/L. Together with the attenuated lesion phenotypes observed after CDOS pretreatment, these results indicate that CDOS acts primarily through host immune activation rather than direct pathogen inhibition, which is consistent with the general mode of action of oligosaccharide elicitors (Sun et al., 2025). At the transcriptional level, CDOS specifically increased the relative expression levels of the SA-associated marker genes *PR1*, *PR2*, and *PR5*, whereas the JA-associated marker genes *VSP* and *PDF1.2* showed no significant changes. In addition, the loss of protection in sid2 and NahG plants, together with the retained protective effect in jar1, supports the interpretation that functional SA signaling is required for CDOS-induced resistance, whereas an intact JA pathway is not essential under the present experimental conditions. This characteristic distinguishes CDOS from some broader-spectrum elicitors that can involve both SA- and JA-related defenses (Yin et al., 2016; Herold et al., 2024).

The H_2_DCFDA fluorescence assay further suggested that CDOS promotes ROS accumulation in *A. thaliana* tissues, supporting the involvement of ROS-associated early signaling in the induced immune response (Wu et al., 2023). Consistent with this observation, CDOS treatment at 50 mg/L increased the activities of POD, PAL, SOD, and CAT, suggesting coordinated activation of downstream defense metabolism and redox regulation. Enhanced POD and PAL activities may contribute to cell-wall reinforcement and phenylpropanoid-associated defense, whereas increased SOD and CAT activities may help maintain redox homeostasis by limiting excessive oxidative damage (Feduraev et al., 2021; Yang et al., 2022). This coordinated sequence of ROS-associated signaling and defense-enzyme activation highlights a multilayered adaptive response to CDOS elicitation (Saha et al., 2023).

The innovation of the present study should also be understood in the context of other curdlan-derived materials. Chemically modified curdlan derivatives, such as oxidized and carboxymethylated curdlan derivatives, have been investigated to improve water solubility and broaden potential applications (Gao et al., 2024). In contrast, the CDOS investigated here were generated by enzymatic hydrolysis without introducing additional chemical substituents, thereby preserving the native β-1,3-glucan oligosaccharide backbone. This feature is advantageous for mechanistic studies because it reduces structural complexity and allows the observed immune-inducing activity to be more directly attributed to CDOS itself. Compared with previous potato-based studies on curdlan oligosaccharides, the present work extends the analysis to the *A. thaliana*–Pst DC3000 pathosystem and provides mutant-supported evidence that the induced resistance is closely associated with SA-related signaling (Li et al., 2014).

Several aspects of the present study still warrant further investigation. The defense-enzyme and gene-expression analyses were conducted only at the selected working concentration of 50 mg/L, which was chosen because it showed the strongest protective effect in the preliminary concentration-screening assay; therefore, the dose–response relationships of these mechanistic readouts remain unresolved. Recent studies have shown that oligosaccharin-induced immune responses can exhibit dose dependence in plants (Wang et al., 2024). In addition, ROS detection relied on H_2_DCFDA fluorescence imaging, and central SA regulatory components such as *NPR1* were not examined. The effects of different CDOS concentrations on plant growth-related traits were also not systematically evaluated. These points do not change the main conclusion that CDOS can function as a plant immune elicitor under the tested conditions, but they do define important directions for future work aimed at strengthening the mechanistic framework and clarifying the biosafety and effective application window of CDOS.

## 5 Conclusion

This study successfully prepared CDOS with a degree of polymerization mainly ranging from 2 to 3 by enzymatic hydrolysis of curdlan. Process and compositional analyses showed a curdlan degradation rate of 31%, an oligosaccharide yield of 23%, a total sugar content of 81.62%, and a residual protein content of 3.25%. Bioactivity assays demonstrated that CDOS did not exhibit direct antibacterial activity against Pst DC3000, but significantly reduced bacterial colonization and alleviated disease symptoms in *A. thaliana* at the selected working concentration of 50 mg/L. The available genetic, transcriptional, and physiological evidence supports that CDOS induces an SA-associated defense response, accompanied by ROS accumulation and increased activities of defense-related enzymes. Overall, these results identify CDOS as a promising plant immune elicitor and provide a useful basis for future studies on its mechanism, biosafety, and potential application in environmentally friendly crop protection.

## Data Availability

The raw data supporting the conclusions of this article will be made available by the authors, without undue reservation.
